# Effect of Low Tube Voltage (100 kV) Combined with ASIR-V on the Visualization and Image Quality of the Adamkiewicz Artery: A Comparison with 120 kV Protocol

**DOI:** 10.3390/diagnostics13152495

**Published:** 2023-07-27

**Authors:** Jiantao Deng, Ting Ma, Jing Yan, Siyi Wu, Gaowu Yan, Hongwei Li, Yong Li, Linwei Zhao, Xiaoping Fan, Morgan A. McClure, Anup Bhetuwal

**Affiliations:** 1 Department of Radiology, Suining Central Hospital, Suining 629000, China; 2 Department of Radiology, The Third Hospital of Mianyang and Sichuan Mental Health Center, Mianyang 621000, China; 3 Department of Radiology and Imaging, Institute of Rehabilitation and Development of Brain Function, The Second Clinical Medical College of North Sichuan Medical College Nanchong Central Hospital, Nanchong 637000, China; 4 Sichuan Key Laboratory of Medical Imaging and Department of Radiology, Affiliated Hospital of North Sichuan Medical College, Nanchong 637000, China

**Keywords:** low tube voltage, iterative reconstruction algorithm, Adamkiewicz artery, computed tomographic angiography

## Abstract

Objective: To evaluate the effect of low tube voltage (100 kV) combined with adaptive statistical iterative reconstruction-V (ASIR-V) on the visualization and image quality of the Adamkiewicz artery (AKA). Methods: One hundred patients were prospectively enrolled and randomly assigned into two groups (both *n* = 50). Group A (100 kV) was reconstructed with filtered back projection (FBP) and ASIR-V from 10% to 100% with 10% intervals. Group B (120 kV) was only reconstructed with FBP. The objective image quality was evaluated by using CT values of the aorta (CT_Aorta_), background noise, signal-to-noise ratio of the descending aorta (SNR_Aorta_), and contrast-to-noise ratio of the spinal cord (CNR_Spinal cord_). The subjective image quality and visualization scores of the AKA were assessed on a 5-point scale. Results: CT_Aorta_ was significantly higher in Group A than in Group B (*p* < 0.001). When ASIR-V weights were ≥60%, significant differences were found in the background noise, SNR_Aorta_, and CNR_Spinal cord_ between the two groups (all *p* < 0.05). In Group A, compared with FBP, the subjective score gradually increased as ASIR-V increased to 80%, which decreased when ASIR-V exceeded 80%. The visualization scores of the AKA (≥60%) and the ability to detect vessel continuity (≥80%) gradually increased as the ASIR-V weights increased (*p* < 0.05). The effective radiation dose was reduced by about 40.36% in Group A compared to Group B. Conclusions: compared with conventional scanning protocol, using a combination of low tube voltage (100 kV) and 80% ASIR-V protocol could not only increase the visualization of the AKA, but also improve image quality and reduce the radiation doses.

## 1. Introduction

The Adamkiewicz artery (AKA), which is also known as the great anterior radiculomedullary artery, was first described in 1882 by Albert Wojciech Adamkiewicz [1]. The AKA originates from the inferior branches of the intercostal or lumbar arteries and connects to the anterior spinal artery (ASA) at the lower 1/3 of the spinal cord. The ASA is the most important artery supplying the thoracolumbar segment of the spinal cord [1,2,3]. During the treatment of thoracic or abdominal aorta diseases, injury or interruption of the AKA and its associated segmental spinal arteries may result in severe spinal cord ischemia. This may introduce two serious postoperative complications: paraplegia or paralysis [4,5,6]. Therefore, the location of the AKA must be considered in some surgical specialties including vascular surgery (e.g., repair of thoracic or abdominal aorta aneurysms), orthopedic surgery (e.g., thoracolumbar spine surgeries), and neurosurgical surgery (e.g., intramedullary tumor resections). As a result, visualization of the local anatomical details and precise localization of the AKA before some surgeries are essential for understanding the blood supply of the thoracolumbar spinal cord and its anatomic variants. It helps a surgeon to formulate a perfect surgical plan, reduce the possibility of damaging the spinal cord blood supply during a surgery, and avoid postoperative complications caused by local spinal cord ischemia [7,8,9].

Intra-arterial computed tomographic angiography (IA-CTA), selective spinal digital subtraction angiography (SSDSA), magnetic resonance angiography (MRA), and conventional CTA all can be used to provide the anatomical details of the AKA [4,5,6,7,10,11]. However, both IA-CTA and SS-DSA are invasive examinations which are complex, technically demanding, and time-consuming, and they may also pose potential risks to patients such as transient cerebral ischemia and transient low paraplegia caused by spinal cord ischemia. Consequently, neither IA-CTA nor SS-DSA should be considered as the first choice for identifying the AKA. MRA is non-invasive and can clearly visualize the AKA with a detection rate of 67% to 93% [5,10,11]. However, due to its limited field of view (FOV), MRA may not visualize the collateral circulation pathways outside the FOV and the openings of the intercostal or lumbar arteries connected to the AKA [5,10,11]. In addition, MRA is also time-consuming and has low spatial resolution, which cannot be widely used because most patients with aortic diseases have an acute and rapid onset. These are serious conditions which prevent successfully completing MRA examinations. With the continuous development of multi-detector computed tomography (MDCT) techniques, there are increasing publications to support the application of aortic CTA for detecting the AKA [12,13,14,15,16]. Previously, two studies have shown that thoracoabdominal aorta CTA with low tube voltage could improve vascular enhancement, AKA visualization, and reduce the radiation dose to patients [12,17]. These CTA protocols, though, could increase background noise to some extent. Therefore, methods for improving AKA visualization and reducing the radiation dose while maintaining image quality is still a clinically meaningful topic. The multi-model-based adaptive statistical iterative reconstruction-V (ASIR-V) technique is a novel and advanced iterative reconstruction method. It can reduce image noise in low-dose acquisitions to provide detailed anatomic information and higher diagnostic accuracy than conventional ASIR [18,19].

In recent years, low tube voltage combined with ASIR-V scanning protocol has been applied to CTA at several sites/organs such as the head and neck [20], coronary arteries [21], portal veins [22], and renal arteries [23]. However, the application of this scanning protocol in preoperative CTA of the aorta and AKA in patients with aortic diseases has not been reported to date. Therefore, the aims of this study were: (a) to compare the ability of CTA with low tube voltage (100 kV) and conventional tube voltage (120 kV) in identification of the AKA and (b) to analyze the effect of low tube voltage (100 kV) combined with the ASIR-V technique on image quality of one-stop-shop CTA images of the aorta and AKA, so as to obtain optimal ASIR-V weights for identifying the AKA without compromising clinical diagnosis.

## 2. Materials and Methods

### 2.1. Study Population

This prospective study was approved by the Ethics Committee of our hospital. All patients were provided with written informed consent forms after understanding the purpose and process of this study.

**Figure 1:**
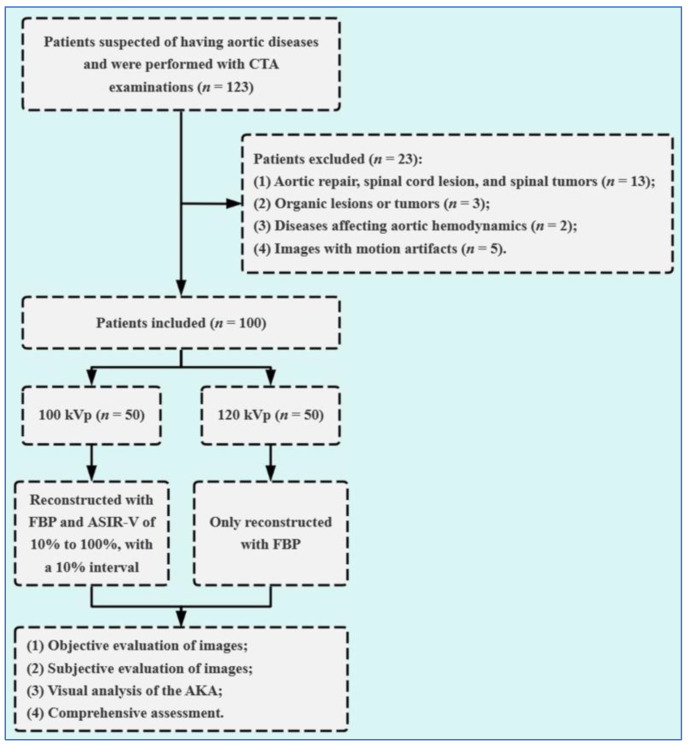
Flow diagram of this study. AKA = Adamkiewicz artery; CTA = computed tomographic angiography; FBP = filtered back projection; ASIR-V = adaptive statistical iterative reconstruction-V.

A total of 123 consecutive patients who had undergone thoracoabdominal aorta CTA scans for the detection of the AKA between January 2021 and January 2022 were included. The conventional 120 kV protocol has been used as the standard protocol for thoracoabdominal aorta CTA at our hospital since the installation of the CT equipment in 2019. The low tube voltage (100 kV) protocol was officially launched at our hospital in June 2021. The inclusion criteria were (1) patients who had undergone thoracoabdominal aorta CTA with a 100 kV or a 120 kV protocol on the same equipment and used the same injection protocol to evaluate the aorta and AKA; (2) were more than 18 years old. The exclusion criteria were (1) patients who had a history of aortic repair, spinal cord lesion, and spinal tumors; (2) patients who had severe organic lesions in the thorax and abdomen or other tumors; (3) patients who had diseases affecting aortic hemodynamics such as hypertension or coronary artery disease; and (4) patients whose images were affected by motion artifacts. A total of 23 patients were excluded. Finally, 100 patients were included in the analysis, and they were randomly divided into Group A (100 kV protocol) and Group B (120 kV protocol) with 50 patients in each group (Figure 1). There were 28 males and 22 females in Group A with a mean age of 65.91 ± 11.19 years (rang 45–74 years), and there were 30 males and 20 females in Group B with a mean age of 65.78 ± 10.69 years (range 42–75 years).

### 2.2. Data Acquisition

On a 256-row CT scanner (Revolution CT; GE Healthcare, Waukesha, WI, USA), patients in Group A underwent 100 kV CT scanning protocol, and patients in Group B underwent 120 kV scanning protocol. Other imaging scanning parameters were the same between both groups: rotation time = 0.5 s; detector coverage = 80 mm; pitch = 0.992:1; coverage speed = 158.75 mm/s; mA mode = manual 375 mA; thickness = 0.625 mm; interval = 0.300 mm; reconstruction kernel = standard; and matrix = 512 × 512. Patients were asked to lie in a supine position with their hands placed on either side of the head and extended. A 20 G intravenous cannula was inserted into the right median cubital vein. Patients in both groups received 400 mg/mL Iomeron (Bracco Sine Pharmaceutical Co., Ltd., Shanghai, China). A total of 90 mL of this contrast agent was injected using a double-barreled high-pressure syringe (Stellant D-CE, Bayer, Germany) at a rate of 4.5 mL/s, followed by a 40 mL saline flush at the same rate. The bolus-tracking technique was used to monitor the central region of the descending aorta at the level of the 9th thoracic vertebra with a region of interest (ROI) size of approximately 100 mm^2^. Threshold monitoring was started 10 s after injection of the contrast agent, and the scan was automatically triggered after a delay of 18 s when the threshold value reached 150 HU. CT scans were obtained from the thoracic inlet to the inferior border of the pubic symphysis. Data were obtained in a head-to-foot direction during a single breath-hold. The volumetric CT dose index (CTDIvol, mGy) and dose-length product (DLP, mGy.cm) after scanning were recorded for each patient, and the effective radiation dose (ED, mSv) was calculated as ED = DLP × 0.015 mSv/mGy.cm.

### 2.3. Image Reconstruction

Axial image reconstruction using acquired raw data was performed with a slice thickness of 0.625 mm, an interval of 0.3 mm, and FOV of 150 mm. In Group A, images were first reconstructed with a filtered back projection (FBP) algorithm (i.e., 0% ASIR-V), and then reconstructed with ASIR-V from 10% to 100% with a 10% interval. A total of 11 sets of images were generated. In Group B, the images were only reconstructed with FBP (i.e., 0% ASIR-V). All reconstructed data were transferred to a picture archiving and communication system (PACS) and RAW 4.7 post-processing workstation for further analysis.

### 2.4. Objective Image Analysis

All objective data were evaluated on a RAW 4.7 post-processing workstation by two radiologists (with 10 and 15 years of experience in reading CTAs) after discussion. A circular ROI was manually placed in the aortic lumen at the level of the 8th, 10th, and 12th thoracic vertebrae on 0.625 mm axial thin-section images. The ROI should cover as much of the aortic lumens as possible, but care should be taken to avoid areas of lesions and beam-hardening artifacts. CT and SD values of the aorta were measured, and mean values were calculated. An ROI of approximately 20 mm^2^ was placed in the spinal cord at the level of the 12th thoracic vertebra, and the CT and SD values of the spinal cord were measured, avoiding the areas of inhomogeneous density and blood vessels that could be observed during the measurement. The SD value of the spinal cord was used as the background noise. Data were measured in three consecutive adjacent levels, and the mean value of ROI in the three levels was taken as the final reference value. The contrast-to-noise ratio (CNR) and signal-to-noise ratio (SNR) were calculated by using the following equations:

SNR_Aorta_ = CT_Aorta_/SD_Aorta_, CNR_Spinal cord_ = (CT_Aorta_ − CT_Spinal cord_)/SD_Spinal cord_.

There are two reasons why we evaluated the degree of enhancement of the AKA by measuring the CT value of the descending aorta [24]. On one hand, the diameter of the AKA is too small, and it has a tortuous path. Therefore, the CT value of the AKA could not be accurately measured. By contrast, the degree of enhancement of the descending aorta is relatively constant under the same scanning protocol. Thus, the measurement of CT value of the descending aorta is relatively stable. On the other hand, the AKA and ASA originate from the intercostal or lumbar arteries which are considered to have similar degrees of enhancement to the descending aorta.

### 2.5. Subjective Scoring of Image Quality

Twelve sets of axial images were scored subjectively by two independent, blinded radiologists (with 12 and 18 years of experience in reading CTAs) who used a 5-point scale. The scoring criteria was as follows: 5 points = the image quality is very good, with very clear visualization of the anatomical details and no obvious background noise; 4 points = the image quality is good, with clear visualization of the anatomical details and less background noise; 3 points = the image quality is fair, and the anatomical details are generally visualized with acceptable background noise; 2 points = the image quality is poor, and the anatomical details are poorly visualized with obvious background noise; and 1 point = the image quality is very poor, and the anatomical details are indistinguishable with considerable background noise. In case of disagreement, consensus was reached through consultation.

### 2.6. Visual Assessment of the AKA

**Figure 2:**
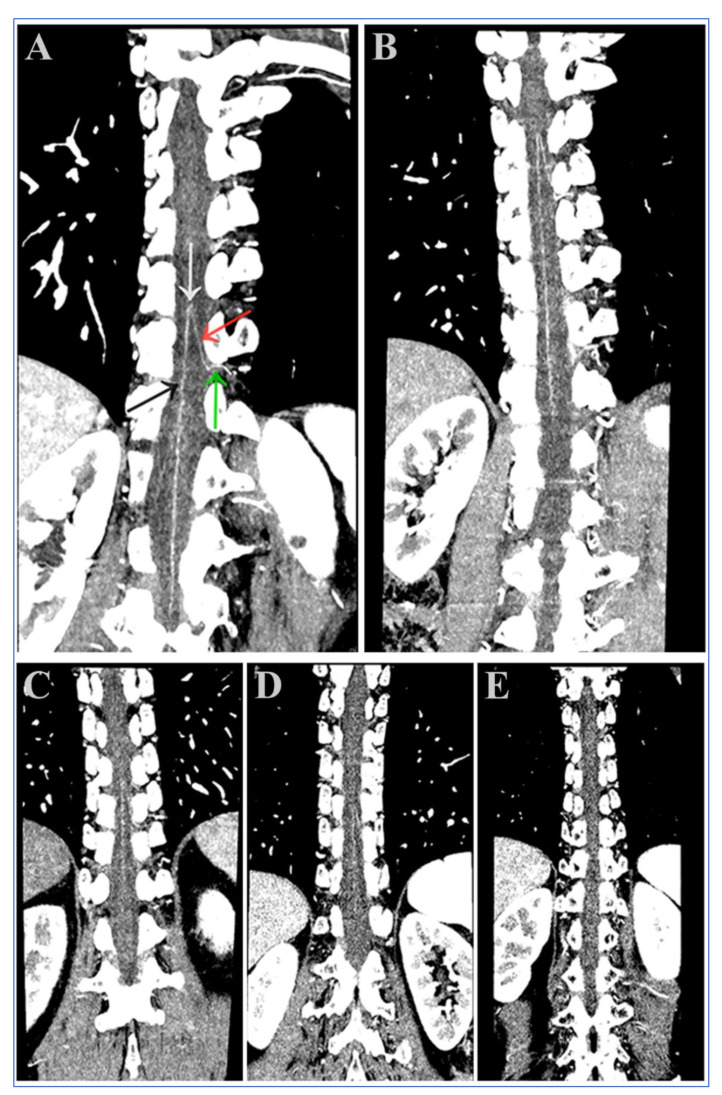
(**A**–**E**) Oblique coronal multiplanar reconstruction images along the aorta. The visualization scores of the AKAs were 5 (**A**), 4 (**B**), 3 (**C**), 2 (**D**), and 1 (**E**), respectively. The black arrow indicates the anterior spinal artery (ASA); the red arrow indicates the Adamkiewicz artery (AKA); the green arrow indicates the radiculomedullary artery (RMA); and the white arrow indicates the characteristic hairpin turn (**A**).

Maximum intensity projection (MIP) and multiplanar reformation (MPR) post-processing were performed on a post-processing workstation by the two radiologists mentioned above. They demonstrated oblique coronal images along the course of the ASA and AKA to show a characteristic hairpin turn. The visualization of the AKA was assessed by using the 5-point scale proposed by Nishida et al. [25,26]. The scoring criteria was as follows (Figure 2): 5 points (excellent) = there is no interruption between the ASA and AKA as well as its origins at the intercostal or lumbar arteries. The entire course of the spinal artery can be clearly visualized. The characteristic hairpin turn formed at the union of the AKA and ASA can also be clearly visualized (white arrow); 4 points (fine) = partial blurring between the AKA and its origins at the intercostal or lumbar arteries at the intervertebral foramen (red arrow) can be observed without discontinuity. The entire course of the spinal artery can be clearly visualized; 3 points (good) = the hairpin turn is clearly visualized. The continuity between the AKA and ASA and its origins at the intercostal or lumbar artery (green arrow) as well as the entire course of the spinal artery is basically visible which just meets the basic diagnostic requirements; 2 points (fair) = although the appearance of the characteristic hairpin turn can be identified (white arrow), the continuity between the AKA and its origin at the intercostal or lumbar arteries cannot be identified; and 1 point (poor) = the characteristic hairpin turn cannot be visualized exactly, and it could not be used for diagnosis.

The visualization scores of the AKA were evaluated randomly and independently by the two radiologists mentioned above who were blinded to patients’ information and the scanning protocol. During the scoring process, agreement was reached by the two radiologists after discussion, and a score of 3 and above could be used for clinical diagnostic analysis. Additionally, the level of origin and course of the AKA from the segmental arteries were also analyzed.

### 2.7. Statistical Analysis

Statistical analyses were performed with software SPSS, version 20.0 (Statistical Package for the Social Sciences, IBM Corp., Armonk, NY, USA). Differences were considered statistically significant at *p* < 0.05. Continuous variables were expressed as mean ± standard deviation (SD) or median and range, and categorical variables were expressed as percentages (%), as appropriate. An independent sample *t* test was used to compare the differences in patients’ age, height, and weight between groups. A Chi-square (*χ*^2^) test was used to compare the differences in patients’ sex, type of aortic diseases, the detection rate of the AKA, and the ability to detect vessel continuity between groups. A *Kruskal-Wallis H* test was used to compare the differences in CT value, background noise, CNR, SNR, and subjective image quality scores. The Bonferroni method was used for multiple comparisons. The Mann–Whitney U test was used to compare the differences in the radiation dose indicators and the visualization scores of the AKA between groups. The *Kappa* test was used to determine the agreement of subjective scores between the two radiologists. A *Kappa* value of 0.75 to 1.0 indicated excellent agreement; a Kappa value of 0.40 to 0.75 indicated intermediate agreement; and a Kappa value of 0.0 to 0.40 indicated poor agreement.

## 3. Results 

### 3.1. Demographic and Clinical Characteristics of Patients

There were no statistically significant differences between the two groups in terms of demographic and clinical characteristics, such as age, height, weight, and sex (*p* > 0.05, Table 1). The diagnoses included aortic dissection (AD, *n* = 27), aortic aneurysm (AA, *n* = 24), aortic intermural hematoma (IMH, *n* = 21), penetrating atherosclerotic aortic ulcer (PAU, *n* = 17), and normal (*n* = 11).

**Figure 3:**
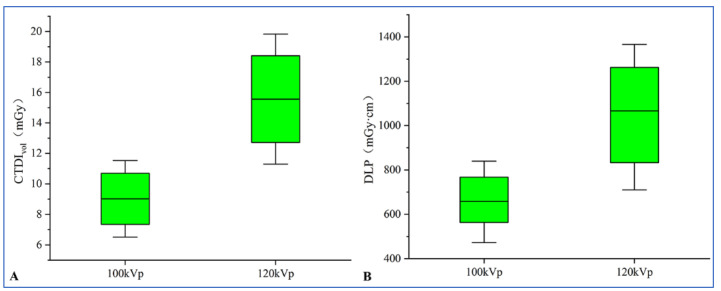
CTA at 100 kV yields significantly lower CTDIvol (**A**) and DLP (**B**) compared with 120 kV (both *p* < 0.001). Boxes represent the first and third quartiles, and whiskers extend to the 5th and 95th percentiles. CTA = computed tomographic angiography; CTDIvol = volumetric computed tomography dose index; and DLP = dose-length product.

The radiation dose indicators in Group A significantly decreased compared with Group B ((median CTDIvol: 8.91 mGy (range, 7.67–10.51 mGy) vs. 16.09 mGy (range, 12.39–17.73 mGy), *p* < 0.001); median DLP: 555.55 mGy.cm (range, 649.41–770.89 mGy.cm) vs. 1088.73 mGy.cm (range, 832.62–1262.55 mGy.cm), *p* < 0.001); median ED: 9.74 mSv (range, 8.33–11.56 mSv) vs. 16.33 mSv (range, 12.48–18.94 mSv), *p* < 0.001, Figure 3)).

### 3.2. Objective Evaluation of Images

**Figure 4:**
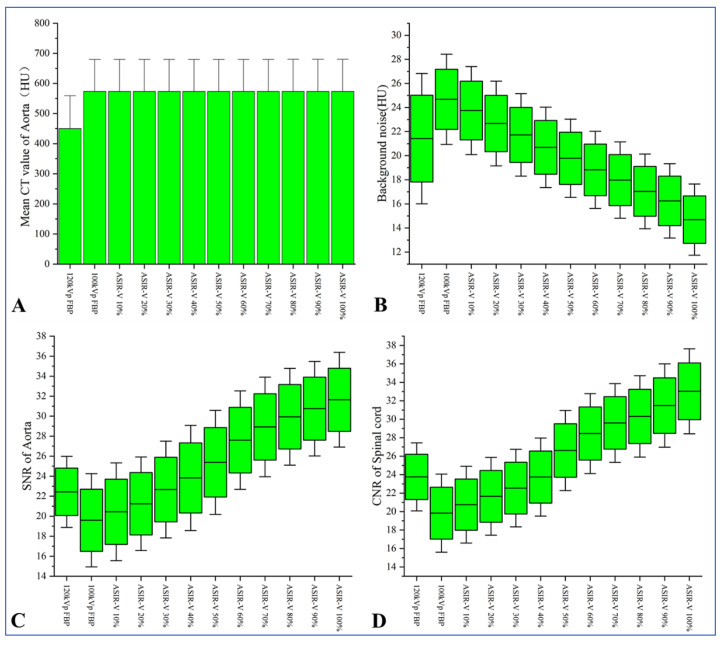
(**A**) Bar graph shows that the mean CT value of the aorta was significantly higher in Group A compared to Group B (*p* < 0.05). Data presented as the mean (boxes) and error of mean (whiskers). (**B**–**D**) Box plots showing that as ASIR-V weights increased, the background noise gradually decreased, while the SNR of the aorta and the CNR of the spinal cord gradually increased in Group A. The boxes represent the first and third quartiles, and whiskers extend to the 5th and 95th percentiles. ASIR-V = adaptive statistical iterative reconstruction-V; CNR = contrast-to-noise ratio; and SNR = signal-to-noise ratio.

Comparison of objective image parameters between groups A and B under different reconstruction algorithms is shown in Table 2. The CT value of the aorta was significantly higher in Group A compared to Group B (*p* < 0.05), but no statistically significant difference in the CT value of the aorta was found in group A under different ASIR-V conditions (*p* > 0.05). As the ASIR-V weights increased, the background noise gradually decreased, while the SNR of the aorta, and the CNR of the spinal cord gradually increased in Group A. Bonferroni multiple comparison tests showed that when ASIR-V was more than 60%, statistically significant differences were found in the background noise, SNR_Aorta_, and CNR_Spinal cord_ between Group A and Group B (background noise, *p* = 0.047; SNR_Aorta_: *p* < 0.001; CNR_Spinal cord_: *p* < 0.001, Figure 4).

### 3.3. Subjective Evaluation of Images

A comparison of subjective image quality scores between Group A and Group B under different reconstruction algorithms is shown in Table 3. The agreement between the two radiologists for subjective evaluation was moderate to excellent (Kappa = 0.679 to 0.909). In Group A, compared with FBP, the subjective scores gradually increased as ASIR-V increased to 80%; however, the subjective scores decreased when ASIR-V exceeded 80%. The subjective scores were higher with 50–100% ASIR-V compared to Group B. Bonferroni multiple comparison tests showed that the subjective scores with 50% ASIR-V and 60% ASIR-V in Group A were not statistically different from those of Group B (*p* = 1.000 and *p* = 0.323, respectively). Whereas statistically significant differences were found between 70% and 100% ASIR-V in Group A compared to Group B (*p* < 0.05). Additionally, in Group A, 80% ASIR-V showed the highest subjective score, and there were statistically significant differences in the subjective scores between 80% ASIR-V and 70% and 90% ASIR-V (both *p* values < 0.05).

### 3.4. Visual Analysis of the AKA

**Figure 5:**
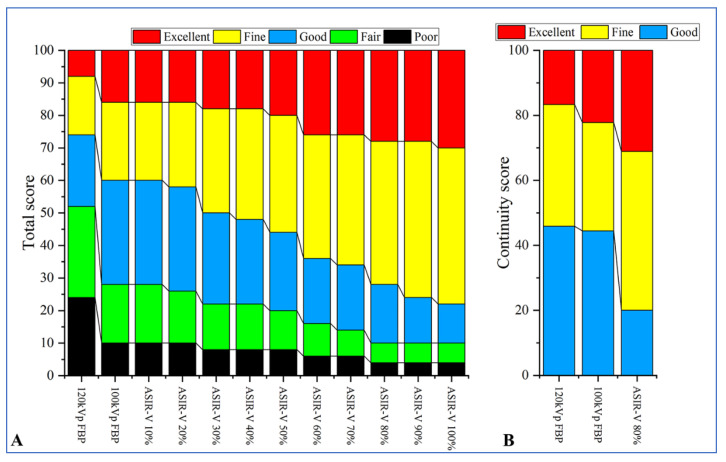
The differences in visualization score of the AKA (**A**) and vessel continuity score (**B**) between the two groups. The 100 kV group with 80% ASIR-V had a significantly higher visualization score compared to Group B (120 kV with FBP) and also had a significantly higher continuity score compared to the 100 kV with FBP group. ASIR-V = adaptive statistical iterative reconstruction-V; AKA = Adamkiewicz artery; and FBP = filtered back projection.

Comparison of the visualization scores of the AKA between Group A and Group B under different reconstruction algorithms is shown in Table 4 and Figure 5. The agreement between the two radiologists for evaluation of visualization scores was excellent (Kappa = 0.842, *p* < 0.001). There were statistically significant differences in the visualization scores of the AKA between Group A and Group B (*p* < 0.001). The total visualization scores of the AKA in 100 kV with FBP was 3.18 ± 1.21 which was higher than that of Group B (3.18 ± 1.21 vs. 2.58 ± 1.26, *p* = 0.017). In Group A, as ASIR-V weights increased and the visualization scores of the AKA and the ability to detect vessel continuity gradually increased. The Mann–Whitney U test showed that in Group A, the visualization scores of the AKA were higher in 60–100% ASIR-V compared to 100 kV with FBP (*p* = 0.029), but AKA visualization scores in 60% ASIR-V was not statistically different from that of 100% ASIR-V (*p* = 0.243). Visualization scores of 80–100% ASIR-V showed a higher ability to detect vessel continuity compared to 100 kV with FBP (*p* = 0.020), but the differences were not statistically significant when ASIR-V was greater than 80% (*p* = 1.000).

**Figure 6:**
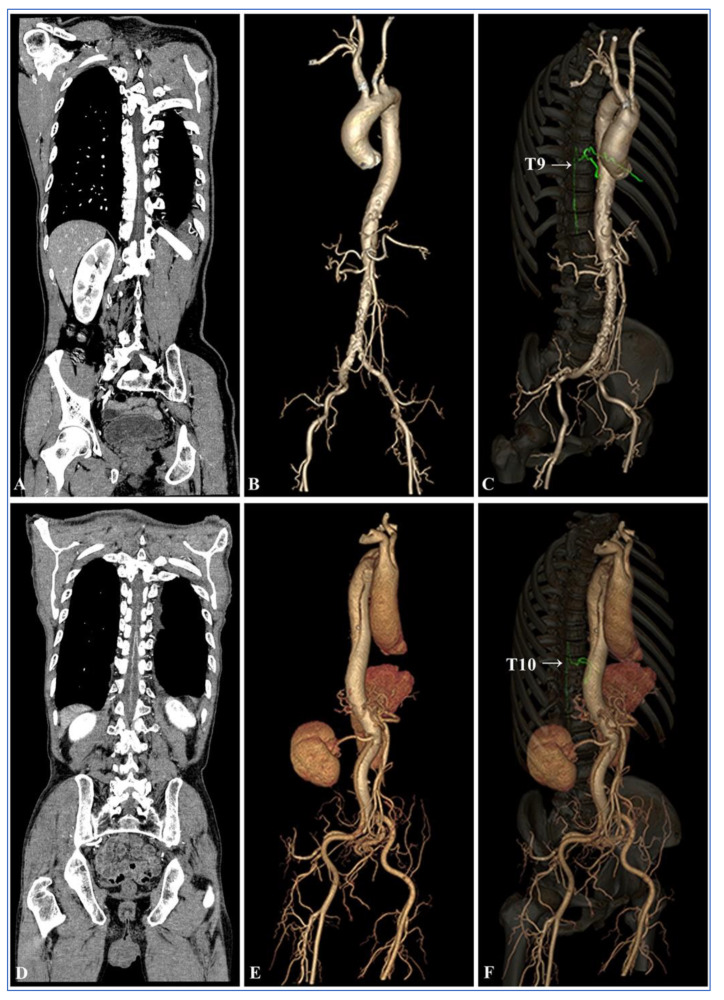
(**A**–**C**) Thoracoabdominal aorta CTA images reconstructed with 120 kV and FBP in a 66-year-old male patient. (**A**) The subjective image quality score of oblique multiplanar reconstruction images obtained along the course of ASA and AKA was 4, and the visualization score of the AKA was 4; (**B**) VR shows multiple atheromatous plaques in the aorta with the formation of penetrating atherosclerotic ulcer. (**C**) The 3D VR image in the right anterior oblique view shows the continuity between the AKA and the aorta, ASA, and the AKA originated at the level of the 9th intercostal artery. (**D**,**E**) Thoracoabdominal aorta CTA reconstructed with 100 kV and FBP in a 75-year-old male patient. (**D**) The subjective image quality score of oblique multiplanar reconstruction images obtained along the course of the ASA and AKA was 3, and the visualization score of the AKA was 5; (**E**) VR shows a DeBakey Type III aortic dissection with intimal tear at the aortic arch and the involvement of the right internal iliac artery; and (**F**) 3D VR image in the right anterior oblique view shows the continuity between the AKA and the aorta, ASA, and the AKA originated at the level of the 10th intercostal artery. CTA = computed tomographic angiography; ASA = anterior spinal artery; FBP = filtered back projection; AKA = Adamkiewicz artery; and VR = volume rendering.

**Figure 7:**
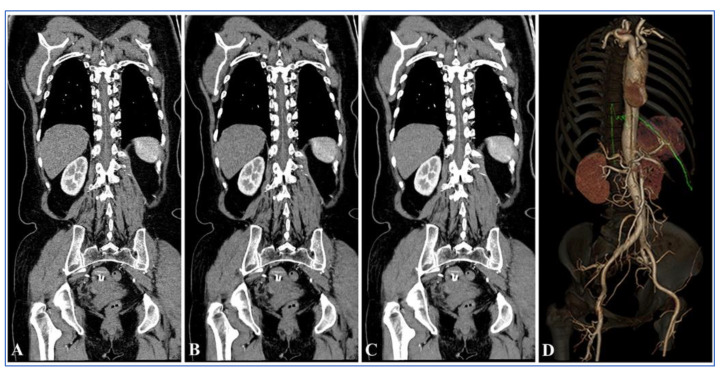
Thoracoabdominal aorta CTA at 100 kV in a 60-year-old female patient. (**A**–**C**) Oblique multiplanar reconstruction images obtained along the course of the ASA and AKA. (**A**) For FBP reconstruction, the subjective image quality score was 3, and the visualization score of the AKA was 3. (**B**) For ASIR-V 40% reconstruction, the subjective image quality score was 4, and the visualization score of the AKA was 4. (**C**) For 80% ASIR-V reconstruction, the subjective image quality score was 5, and the visualization score of the AKA was 5. (**D**) The 3D VR image of the right anterior oblique shows the continuity between the AKA and the aorta, ASA, and the AKA originated at the level of the 10th intercostal artery. A Stanford Type A aortic dissection was revealed, and an intimal tear was located at the plane of the aortic arch, which extended downward to the right common iliac artery. CTA = computed tomographic angiography; ASIR-V = adaptive statistical iterative reconstruction-V; ASA = anterior spinal artery; FBP = filtered back projection; AKA = Adamkiewicz artery; and VR = volume rendering.

**Figure 8:**
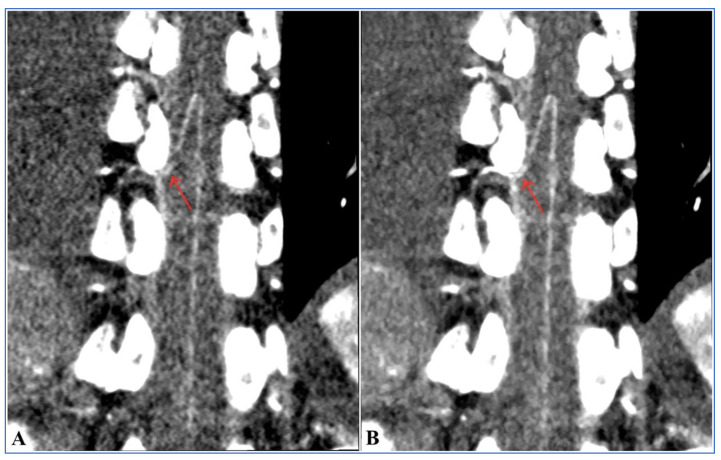
Preoperative 100 kV CTA images of a 55-year-old female patient with aortic aneurysm obtained with a small field of view. (**A**) FBP reconstruction showed high image noise and unsatisfactory visualization of the AKA (red arrow), especially at the intervertebral foramen, with a subjective image quality score of 3 and a visualization score of 3. (**B**) ASIR-V 80% reconstruction showed obviously reduced image noise and significant improvement in visualization of the AKA artery (red arrow) which can also determine the level of origin of the AKA with a subjective image quality score of 5 and visualization score of 5. CTA = computed tomographic angiography; ASIR-V = adaptive statistical iterative reconstruction-V; AKA = Adamkiewicz artery; and FBP = filtered back projection.

In the 100 kV with FBP group, the characteristic hairpin turn was observed in 45 (90.0%) patients, and the continuity between the AKA and intercostal or lumbar arteries was observed in 36 (72.0%) patients. In the 100 kV with 80% ASIR-V group, the characteristic hairpin turn was observed in 48 (96.0%) patients and the continuity between the AKA and intercostal or lumbar arteries were observed in 45 (90.0%) patients. In Group B (120 kV with FBP), the characteristic hairpin turn and the continuity between the AKA and intercostal or lumbar arteries were observed in 38 (76.0%) and 24 (48.0%) patients, respectively. In terms of the ability to detect the characteristic hairpin turn, there were no statistically significant differences between the 100 kV with 80% ASIR-V and 100 kV with FBP groups (96.0% vs. 90%, *p* = 0.433), as well as between the 100 kV with FBP group and Group B (120 kV FBP) (90.0% vs. 76.0%, *p* = 0.062). However, 100 kV with 80% ASIR-V showed a higher ability to detect the characteristic hairpin turn compared to the 120 kV with FBP (96.0% vs. 76.0%, *p* = 0.004). The ability to detect the continuity between the AKA and intercostal or lumbar arteries was higher in the 100 kV with 80% ASIR-V and 100 kV with FBP groups compared to the 120 kV with FBP group (90.0% vs. 48.0%, *p* < 0.001; 72.0% vs. 48.0%, *p* = 0.014, respectively). The 100 kV with 80% ASIR-V protocol showed a higher ability to detect the continuity between the AKA and the intercostal or lumbar arteries compared to 100 kV with FBP (90.0% vs. 72.0%, *p* = 0.022). The images of representative patients are shown in Figure 6, Figure 7 and Figure 8.

### 3.5. Comprehensive Assessment

**Figure 9:**
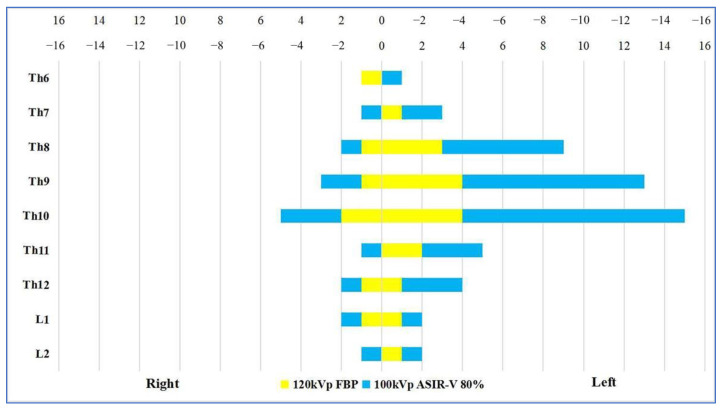
Distribution of the level of AKA origin through the thoracic and lumbar segments in 72 assessable patients. Th = thoracic spine; L = lumbar spine; and AKA = Adamkiewicz artery.

Based on the above-mentioned findings, the use of 80% ASIR-V had higher objective image quality evaluation results, subjective score, and visualization score of the aorta as well as the AKA compared with other groups. Thus, the 100 kV with 80% ASIR-V group and Group B were selected to evaluate the level of origin and course of the AKA from the intercostal or lumbar arteries (Figure 9). Among 100 patients who underwent thoracoabdominal aorta CTA, the AKA was successfully visualized in 72 patients. Out of these 72 patients, the AKA originated from the left intercostal or lumbar arteries in 54 (75.0%) patients, from the right intercostal or lumbar arteries in 18 (25.0%) patients, and it was seen to originate from the 8th intercostal artery to the level of the first lumbar artery in 63 (87.5%) patients.

## 4. Discussion

In this study, it was demonstrated that (a) low tube voltage (100 kV) combined with ASIR-V protocol can be used to visualize the AKA; (b) low tube voltage (100 kV) combined with ASIR-V protocol could not only increase the visualization of the AKA but also improve image quality and reduce the radiation doses; and (c) the 80% ASIR-V reconstruction algorithm achieved the best image quality in the 100 kV AKA CTA. As far as we know, no studies were reported in previous literature in which the researchers had explored the application of low tube voltage (100 kV) combined with the latest ASIR-V protocol for the improvement of AKA visualization.

Previous studies have mainly investigated the optimal contrast injection protocol for the visualization of the AKA [27], data reconstruction [13,25], and the application of ultra-high-resolution CTA in the delineation of the AKA [16,28]. However, the effect of low tube voltage in improving AKA visualization has been rarely studied. Shimoyama et al. [12] found that 70 kV aortic CTA has substantial advantages over 120 kV CTA for the detection of the AKA. However, the scanning protocols (including the CT equipment, contrast injection protocols, and data reconstruction algorithms) were not consistent between the two groups [12]. Kubota et al. [17] found that under identical scanning conditions, the 100 kV scanning protocol was superior to the 120 kV protocol in terms of objective image parameters and visualization scores of the aorta and AKA. Unfortunately, the authors in that study did not adopt low tube voltage scanning protocol with the iterative reconstruction algorithm to improve the image quality of the aorta and AKA [17].

It has been reported that AKA can be successfully visualized when adequate vascular enhancement of the aorta and its inferior branch arteries (e.g., intercostal or lumbar arteries) is achieved and the contrast of the aorta is increased [29,30]. Our study showed that the mean CT value of the aorta in Group A (573.86 HU) was approximately 28.56% higher than Group B (446.36 HU); the total visualization scores of the AKA was higher in the 100 kV with FBP group than in Group B (3.18 ± 1.21 vs. 2.58 ± 1.26, *p* = 0.017); and the detection rate of the continuity between the AKA and segmental arteries (intercostal and lumbar arteries) was higher in the 100 kV with FBP group than in the 120 kV with FBP group (72.0% vs. 48.0%, *p* = 0.032). The results suggest that low tube voltage can enhance the degree of vessel enhancement and improve AKA visualization. This result is consistent with the result of Kubota et al. [17] that 100 kV is better than 120 kV in AKA evaluation. This is due to the fact that, during low tube voltage CT scans, the interaction of X-rays with matter primarily produces a photoelectric effect, and photon energies are closer to the K-edge of iodine-containing tissues (33.2 keV) [31]. This physical phenomenon increases X-ray absorption of intravascular iodinated contrast agents, elevating the CT value of the vessels, and finally improving the contrast of the aorta and AKA to the surrounding tissues.

Our study also showed that the effective radiation dose was reduced by about 40.36% in Group A compared to Group B. The radiation dose is positively correlated with the square of the tube voltage, so even a small reduction in tube voltage can result in a significant reduction in radiation dose [32]. Shimoyama et al. [12] reported that the radiation dose was approximately 66% lower in the 70 kV group compared to the 120 kV group, and the 70 kV protocol could improve the visualization of the arterial continuity without affecting the AKA detection rate. An evaluation on aortic CTA protocols showed that aortic CTA which used low tube voltages (80 to 100 kV) resulted in a 56% decreased radiation dose compared to the 120 kV protocol while maintaining image quality [33]. The results of our study are consistent with previous findings.

Image noise is negatively correlated with the tube voltage [22,32,33,34,35]. As a result, the low radiation dose obtained by using low tube voltage can result in high image noise and poor image quality. The image noise in our study of group B was only slightly lower compared to group A (13.21%). This is because we used a 100 kV tube voltage instead of an 80 kV or 70 kV tube voltage (we were worried that using an 80 kV or 70 kV tube voltage protocol might result in CTA images which could not be interpreted by our radiologist). In a study by Ren et al. [35], the image noise in a group of 80 kV-FBP was also only slightly lower compared to a group of 120 kV-FBP (22.53% for the right renal artery and 22.72% for the left renal artery, respectively). The image noise of our study is consistent with the study by Ren et al. [35].

Our study showed that with the ASIR-V weights increased, the background noise gradually decreased. By contrast, the SNR of the aorta and the CNR of the spinal cord gradually increased in Group A, which indicated that the ASIR-V algorithm could improve the image quality while reducing image noise. These results are similar to the findings from a study on the use of ASIR-V in liver CT conducted by Chen et al. [34]. Our study also showed that reconstruction with 40–100% ASIR-V in Group A had lower background noise and higher CNR and SNR compared to Group B. However, after the Bonferroni multiple comparison tests, it showed that only when the ASIR-V weights were more than 60%, can statistically significant differences in the background noise, CNR, and SNR be found between the two Groups. The results indicated that under the low tube voltage condition, higher ASIR-V weights can significantly reduce the background noise and also significantly improve the CNR and SNR values. As was shown in Group A, compared to reconstruction with FBP, the subjective image quality scores gradually increased as ASIR-V weights increased to 80%, while the subjective image quality scores were reduced when ASIR-V weights were greater than 80%. This may be due to the higher post-ASIR-V weights, which can cause noise frequency changes and drift. This subsequently causes the whole image to become excessively smooth, which ulteriorly leads to distortions and even the occurrence of “plastic texture” in the images; thereby, reducing the subjective image quality scores assessed by radiologists. These results are consistent with the findings from the studies of Ren et al. [22,35].

Our study also demonstrated that reconstruction with 50–100% ASIR-V in Group A showed higher subjective scores compared to Group B. However, after the Bonferroni multiple comparison tests, the subjective scores with 50% ASIR-V and 60% ASIR-V in Group A were not statistically different from those in Group B. On the contrary, statistically significant differences were found between 70–100% ASIR-V for Group B, as well as between 80% ASIR-V and 70% ASIR-V, and 90% ASIR-V. These results indicated that under low tube voltage conditions, images reconstructed with 80% ASIR-V have the best image quality of the aorta and AKA. Some investigators applied ASIR-V in low-dose CTAs of the portal vein, renal artery, and coronary artery, suggesting that the best image quality can be obtained for 80%, 70%, and 100% ASIR-V [23,35,36]. Our results are basically consistent with the above-mentioned findings. The differences in the optimal ASIR-V weights may be due to the difference in imaging sites/organs.

To the best of our knowledge, no studies have explored the application of low tube voltage (100 kV) combined with the latest ASIR-V protocol for the improvement of AKA visualization. Improving the CNR of the spinal cord is essential to improve the detectability and visualization of micro vessels. Previous studies have shown that conventional tube voltage (120 kV) combined with the model-based iterative reconstruction (MBIR) protocol can significantly reduce image noise, improve the SNR of the aorta, and the CNR of the spinal artery compared to the FBP and ASIR algorithms. This has improved the visualization of the AKA and detectability of continuity of the segmental arteries [25,37]. Our results showed that as the ASIR-V weights increase, the visualization scores of the AKA and the ability to detect vessel continuity gradually increase. Additionally, 80% ASIR-V showed significantly higher visualization scores, improving the ability to detect vessel continuity, the SNR of the aorta, and the CNR of the spinal cord compared to 100 kV with FBP. This indicated that the use of a higher ASIR-V algorithm at low tube voltage can significantly improve the visualization of the AKA. The MBIR and ASIR algorithms were not applied or compared in our study. Our reason for the improvements in AKA visualization may be partly due to the fact that ASIR-V, as a new generation of iterative reconstruction algorithms developed in recent years, incorporates object, physical, and noise models as a whole. This algorithm abandons traditional optical models and has greater ability to reduce image noise and artifacts compared to ASIR, and it also has a shorter reconstruction time and better spatial resolution compared to MBIR [38,39]. Therefore, the ASIR-V algorithm may be more useful in the visualization of arterial small blood vessels close to the bone structure and thus improve the visualization of the ASA and AKA.

Our study showed that the AKA originated from the left intercostal artery or left lumbar artery in 75.0% of the patients and originated from the 8th intercostal artery to the level of the first lumbar artery in 87.5% of the patients. The results are consistent with findings from previous studies [12,13,17]. Our study also showed that the continuity between the AKA and intercostal or lumbar arteries were observed in 72.0% of the patients in the 100 kV FBP group (before using the iterative algorithm) and 90% of the patients in the 100 kV ASIR-V 80% group (after using the iterative algorithm), which are slightly higher than previous findings (25% to 71%) [5,17,27,29]. The reasons for this may be as follows: (1) The difference in the CT equipment used among studies. Utsunomiya et al. [29] used a 64-row spiral CT, while our study used a 256-row spiral CT. An increase in spatial resolution may improve the visualization of fine arteries [13]. (2) The difference in the concentration and dosage of the contrast agent administered among studies. The iodine concentration and dose of the contrast agent used by Kubota Y et al. [17] were 350 mg/mL and 70 mL, respectively. Whereas in our study, the iodine concentration and dose of the contrast agent were 400 mg/mL and 90 mL, respectively. An increase in iodine dose may also improve AKA visualization [27]. (3) The difference in the method used to evaluate the visualization of the AKA and its continuity with the intercostal or lumbar artery. Yoshioka K et al. [28] used a 4-point scale, while our study used the 5-point scale proposed by Nishida et al. [25,26]. It is, therefore, difficult to analyze and compare differences in the ability to detect the vessel continuity of the AKA with spinal arteries between the findings of our study and previous studies.

Our study has some limitations. First, this is a single-center study with a relatively small number of patients. Although the findings of our study suggested that the use of low tube voltage combined with a multi-model iterative reconstruction algorithm can improve the image quality and visualization of the AKA, studies with larger patient populations are needed to confirm our findings. Second, the gold standard SS-DSA was not used to validate the identification of the AKA in our study as SS-DSA is an invasive examination, and it is complex, technically demanding, and difficult to perform in patients with aortic diseases, especially those with AD or large AA [40]. In addition, SS-DSA may also pose potential risks to patients with transient cerebral ischemia, renal failure, and transient low paraplegia caused by spinal cord ischemia. Therefore, in order to avoid subjecting patients to unnecessary risks, we no longer perform SS-DSA in such patients. Third, this is a study in an Asian population. The body mass index is smaller in Asian populations compared to some other ethnic groups. Therefore, the applicability of this scanning protocol in patients with larger body mass index is unclear and remains to be confirmed by further studies. Finally, in this study, we only compared the differences between the use of 120 kV and 100 kV tube voltages, and the use of low tube voltages of 80 kV or 70 kV were not analyzed. Low tube voltages may help to reduce the dose of contrast agent used in thoracoabdominal aorta CTAs, which contributes to preventing or reducing the probability of contrast-induced nephropathy in patients with aortic diseases.

In conclusion, our study suggested that the use of low tube voltage (100 kV) combined with 80% ASIR-V in one-stop CTA of the aorta and AKA can, not only increase the sensitivity of the AKA visualization, but also improve image quality that meets clinical requirements and reduce the radiation dose to patents simultaneously.

## Figures and Tables

**Table 1:** Demographics and clinical characteristics of patients in Groups A and B.

Items	Group A (*n* = 50)	Group B (*n* = 50)	*p* Value
Age (years)	65.91 ± 11.19	65.78 ± 10.69	0.928
Height (cm)	164.84 ± 6.77	163.33 ± 6.53	0.284
Weight (kg)	61.62 ± 7.82	61.53 ± 6.40	0.953
Males and Females	28/22	30/20	0.840
Aortic Condition, *n* (%)			
AD	15 (30.0)	12 (24.0)	0.653
AA	11 (22.0)	13 (26.0)	0.815
IMH	12 (24.0)	9 (18.0)	0.624
PAU	7 (14.0)	10 (20.0)	0.595
Normal	5 (10.0)	6 (12.0)	0.749

AD = aortic dissection; AA = aortic aneurysm; IMH = intramural aortic hematoma; and PAU = penetrating atherosclerotic ulcer.

**Table 2:** Comparisons of CT value of the aorta, background noise, SNR, and CNR between Groups A and B.

Groups	Image Reconstruction Methods	CT_Aorta_ (HU)	Background Noise (HU)	SNR_Aorta_	CNR_Spinal cord_
Group B	120 kV FBP	446.36 ± 71.41	21.42 ± 3.61	22.43 ± 2.37	23.76 ± 2.45
Group A	100 kV FBP	573.86 ± 77.01	24.68 ± 2.50	19.59 ± 3.10	19.83 ± 2.81
	100 kV ASIR-V 10%	573.82 ± 77.02	23.75 ± 2.43	20.44 ± 3.26	20.74 ± 2.77
	100 kV ASIR-V 20%	573.83 ± 77.03	22.67 ± 2.34	21.24 ± 3.12	21.65 ± 2.81
	100 kV ASIR-V 30%	573.83 ± 77.04	21.72 ± 2.28	22.66 ± 3.23	22.54 ± 2.80
	100 kV ASIR-V 40%	573.84 ± 77.05	20.69 ± 2.23	23.83 ± 3.50	23.75 ± 2.82
	100 kV ASIR-V 50%	573.84 ± 77.06	19.78 ± 2.17	25.39 ± 3.47	26.61 ± 2.89
	100 kV ASIR-V 60%	573.82 ± 77.10	18.82 ± 2.14	27.60 ± 3.28	28.45 ± 2.88
	100 kV ASIR-V 70%	573.82 ± 77.11	17.97 ± 2.11	28.93 ± 3.32	29.60 ± 2.85
	100 kV ASIR-V 80%	573.83 ± 77.15	17.04 ± 2.07	29.94 ± 3.23	30.31 ± 2.94
	100 kV ASIR-V 90%	573.87 ± 77.16	16.25 ± 2.06	30.75 ± 3.15	31.48 ± 3.01
	100 kV ASIR-V 100%	573.95 ± 77.18	14.69 ± 1.97	31.65 ± 3.16	33.03 ± 3.07
	*H* Value	84.786	386.666	391.562	439.635
	*p* value	<0.001	<0.001	<0.001	<0.001

HU = Hounsfield unit; CNR = contrast-to-noise ratio; SNR = signal-to-noise ratio; ASIR-V = adaptive statistical iterative reconstruction-V; FBP = filtered back projection; CT = computed tomography.

**Table 3:** Comparisons of subjective image quality scores between Groups A and B.

Groups	Image Reconstruction Methods	Radiologist A	Radiologist B	Kappa	*p* Value
Group B	120 kV FBP	3.78 ± 0.48	3.80 ± 0.50	0.909	<0.001
Group A	100 kV FBP	3.06 ± 0.24	3.08 ± 0.27	0.807	<0.001
	100 kV ASIR-V 10%	3.10 ± 0.30	3.08 ± 0.27	0.685	<0.001
	100 kV ASIR-V 20%	3.12 ± 0.33	3.14 ± 0.35	0.719	<0.001
	100 kV ASIR-V 30%	3.26 ± 0.44	3.28 ± 0.45	0.703	<0.001
	100 kV ASIR-V 40%	3.52 ± 0.50	3.56 ± 0.50	0.789	<0.001
	100 kV ASIR-V 50%	3.80 ± 0.40	3.84 ± 0.42	0.875	<0.001
	100 kV ASIR-V 60%	4.16 ± 0.37	4.12 ± 0.33	0.887	<0.001
	100 kV ASIR-V 70%	4.54 ± 0.49	4.50 ± 0.42	0.699	<0.001
	100 kV ASIR-V 80%	4.96 ± 0.20	4.94 ± 0.24	0.847	<0.001
	100 kV ASIR-V 90%	4.48 ± 0.50	4.46 ± 0.48	0.679	<0.001
	100 kV ASIR-V 100%	4.32 ± 0.47	4.30 ± 0.46	0.815	<0.001
	*H* Value	414.257	405.879	-	-
	*p* Value	<0.001	<0.001	-	-

ASIR-V = adaptive statistical iterative reconstruction-V and FBP = filtered back projection.

**Table 4:** Visual assessment of the Adamkiewicz arteries.

Groups	Image Reconstruction Methods	Visualization Scores of the Adamkiewicz Arteries					Hairpin Curve	Branching Level
		1 = Poor	2 = Fair	3 = Good	4 = Fine	5 = Excellent		
Group B	120 kV FBP	12 (24.0%)	14 (28.0%)	11 (22.0%)	9 (18.0%)	4 (8.0%)	38 (76.0%)	24 (48.0%)
Group A	100 kV FBP	5 (10.0%)	9 (18.0%)	16 (32.0%)	12 (24.0%)	8 (16.0%)	45 (90.0%)	36 (72.0%)
	100 kV ASIR-V 10%	5 (10.0%)	9 (18.0%)	16 (32.0%)	12 (24.0%)	8 (16.0%)	45 (90.0%)	36 (72.0%)
	100 kV ASIR-V 20%	5 (10.0%)	8 (16.0%)	16 (32.0%)	13 (26.0%)	8 (16.0%)	45 (90.0%)	37 (74.0%)
	100 kV ASIR-V 30%	4 (8.0%)	7 (14.0%)	14 (28.0%)	16 (32.0%)	9 (18.0%)	46 (92.0%)	39 (78.0%)
	100 kV ASIR-V 40%	4 (8.0%)	7 (14.0%)	13 (26.0%)	17 (34.0%)	9 (18.0%)	46 (92.0%)	39 (78.0%)
	100 kV ASIR-V 50%	4 (8.0%)	6 (12.0%)	12 (24.0%)	18 (36.0%)	10 (20.0%)	46 (92.0%)	40 (80.0%)
	100 kV ASIR-V 60%	3 (6.0%)	5 (10.0%)	10 (20.0%)	19 (38.0%)	13 (26.0%)	47 (94.0%)	42 (84.0%)
	100 kV ASIR-V 70%	3 (6.0%)	4 (8.0%)	10 (20.0%)	20 (40.0%)	13 (26.0%)	47 (94.0%)	43 (86.0%)
	100 kV ASIR-V 80%	2 (4.0%)	3 (6.0%)	9 (18.0%)	22 (44.0%)	14 (28.0%)	48 (96.0%)	45 (90.0%)
	100 kV ASIR-V 90%	2 (4.0%)	3 (6.0%)	7 (14.0%)	24 (48.0%)	14 (28.0%)	48 (96.0%)	45 (90.0%)
	100 kV ASIR-V 100%	2 (4.0%)	3 (6.0%)	6 (12.0%)	24 (48.0%)	15 (30.0%)	48 (96.0%)	45 (90.0%)

ASIR-V = adaptive statistical iterative reconstruction-V and FBP = filtered back projection.

## Data Availability

The data presented in this study are available upon request from the corresponding author (G.Y.). The data are not publicly available due to privacy or ethical concerns.
